# Postoperative Abdominal Myofascial Pain Syndrome in a Cancer Survivor: A Case Report on Diagnostic and Therapeutic Considerations

**DOI:** 10.7759/cureus.96928

**Published:** 2025-11-15

**Authors:** Mayuko Owada, Kumiko Yamada, Yuya Murata, Keiichi Shimazaki, Jungo Kato

**Affiliations:** 1 Department of Anesthesiology, University of Tsukuba Hospital, Tsukuba, JPN; 2 Department of Anesthesiology, Institute of Medicine, University of Tsukuba, Tsukuba, JPN

**Keywords:** abdominal myofascial pain syndrome, abdominal wall pain, cancer survivor, chronic postoperative pain, trigger point injection

## Abstract

Although abdominal myofascial pain syndrome (AMPS) has been reported to cause persistent pain following abdominal surgery, it remains underrecognized, often leading to the development of chronic postoperative pain. We present a case of AMPS characterized by the sudden onset of abdominal pain after esophageal and gastric cancer surgery, which progressed to persistent chronic pain in conjunction with cancer recurrence.

Here, we present the case of a 66-year-old man who underwent subtotal esophagectomy and distal gastrectomy for synchronous esophageal and gastric cancers. Approximately one month after surgery, he developed acute abdominal pain. A diagnosis of AMPS was made based on localized abdominal tenderness with a positive Carnett’s sign and corresponding hyperechoic findings on ultrasound. A trigger point injection (TPI) with 1% lidocaine provided immediate pain relief. However, the pain later recurred at a different site, which was again effectively managed with TPI. As the cancer recurred and progressed, the area of abdominal pain extended, and the TPI efficacy diminished. Due to intractable pain and functional decline, the patient was transitioned to medical management and ultimately to best supportive care.

AMPS can develop acutely in the postoperative course in patients with cancer and may contribute to the development of chronic postoperative pain. Although TPI can be both diagnostically and therapeutically effective, long-term management of AMPS in patients with advanced cancer remains challenging.

## Introduction

Abdominal myofascial pain syndrome (AMPS) is a chronic pain condition characterized by hypersensitive nodules, known as trigger points (TPs), in the abdominal wall muscles, resulting in localized pain and tenderness [1]. It is typically diagnosed clinically and can be differentiated from visceral pain, which originates from internal organs and tends to be dull or diffuse, by a positive Carnett’s sign, in which pain intensifies when the abdominal muscles are tensed [2].

Although AMPS has been reported as a cause of abdominal wall pain following abdominal surgery, it remains frequently under-recognized, leading to misdiagnosis and suboptimal pain management [3]. In postoperative cancer survivors, decreased overall well-being and activity restrictions may precipitate AMPS, creating a vicious cycle in which reduced activity, increased pain, and depression can further exacerbate symptoms [4]. Here, we report a severe case of AMPS that developed after surgery for esophageal and gastric cancer and ultimately progressed to persistent chronic abdominal pain. Written informed consent was obtained from the patient for the publication of this case report. This report presents the clinical course, diagnostic process, and therapeutic management of this case, followed by a discussion of its implications for postoperative pain care.

## Case presentation

A 66-year-old man (height: 164 cm; weight: 41 kg), with concurrent esophageal and gastric cancers, underwent right thoracotomy with subtotal esophagectomy, three-field lymph node dissection, distal gastrectomy, cholecystectomy, and jejunostomy placement. The surgery was successful, and he was discharged uneventfully on postoperative day 14 without significant postoperative pain. However, 27 days later, he experienced the sudden onset of abdominal pain and was urgently readmitted to our hospital the following day. On admission, gastrointestinal endoscopy, abdominal computed tomography, electrocardiogram, and blood tests revealed no major abnormalities. A consultation with the pain clinic was subsequently conducted.

**Figure 1:**
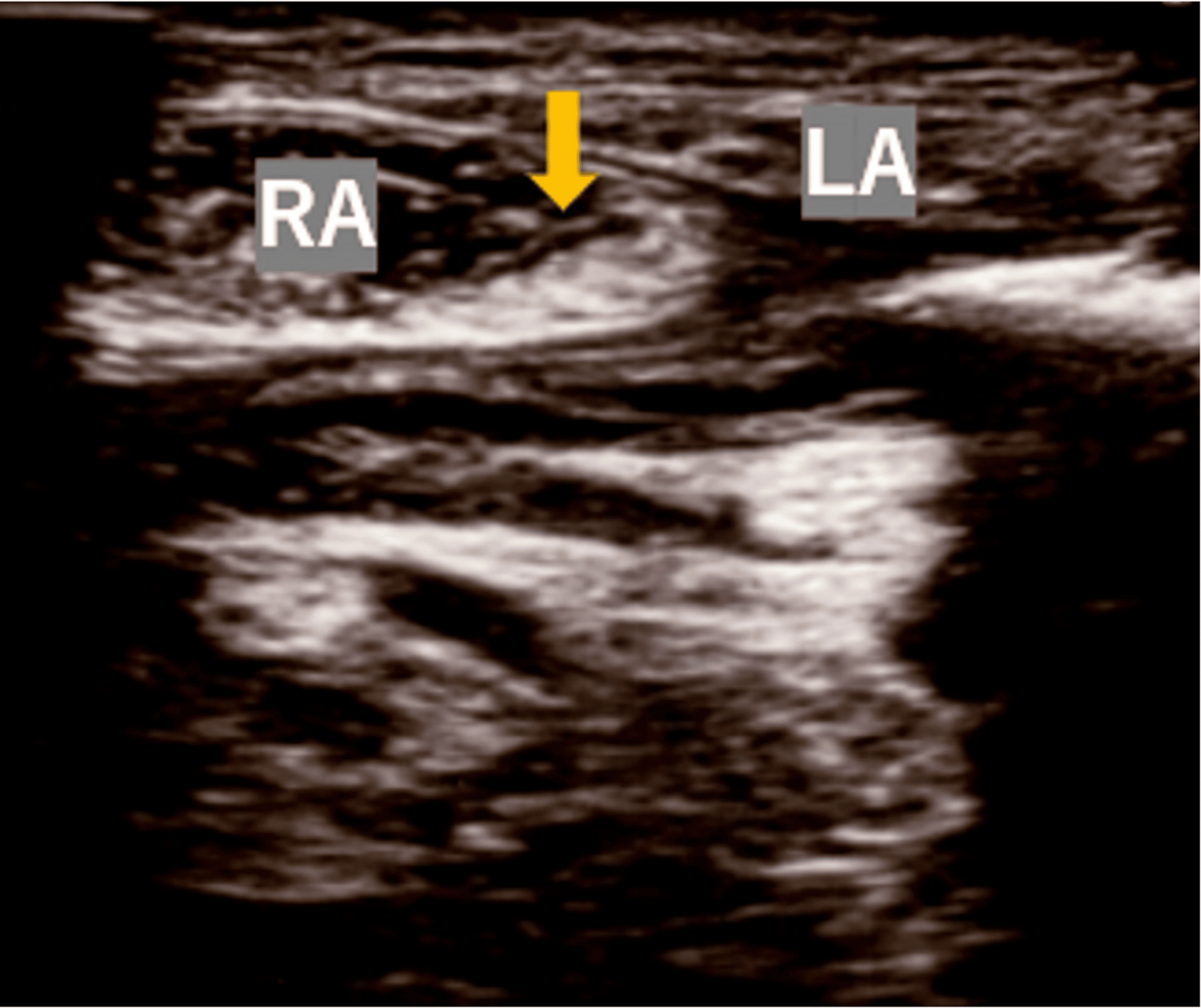
Ultrasound image of the trigger point at the RA muscle on the second visit to our pain clinic. A hyperechoic area (arrow) was identified at the inner edge of the RA muscle LA: linea alba; RA: rectus abdominis

**Figure 2:**
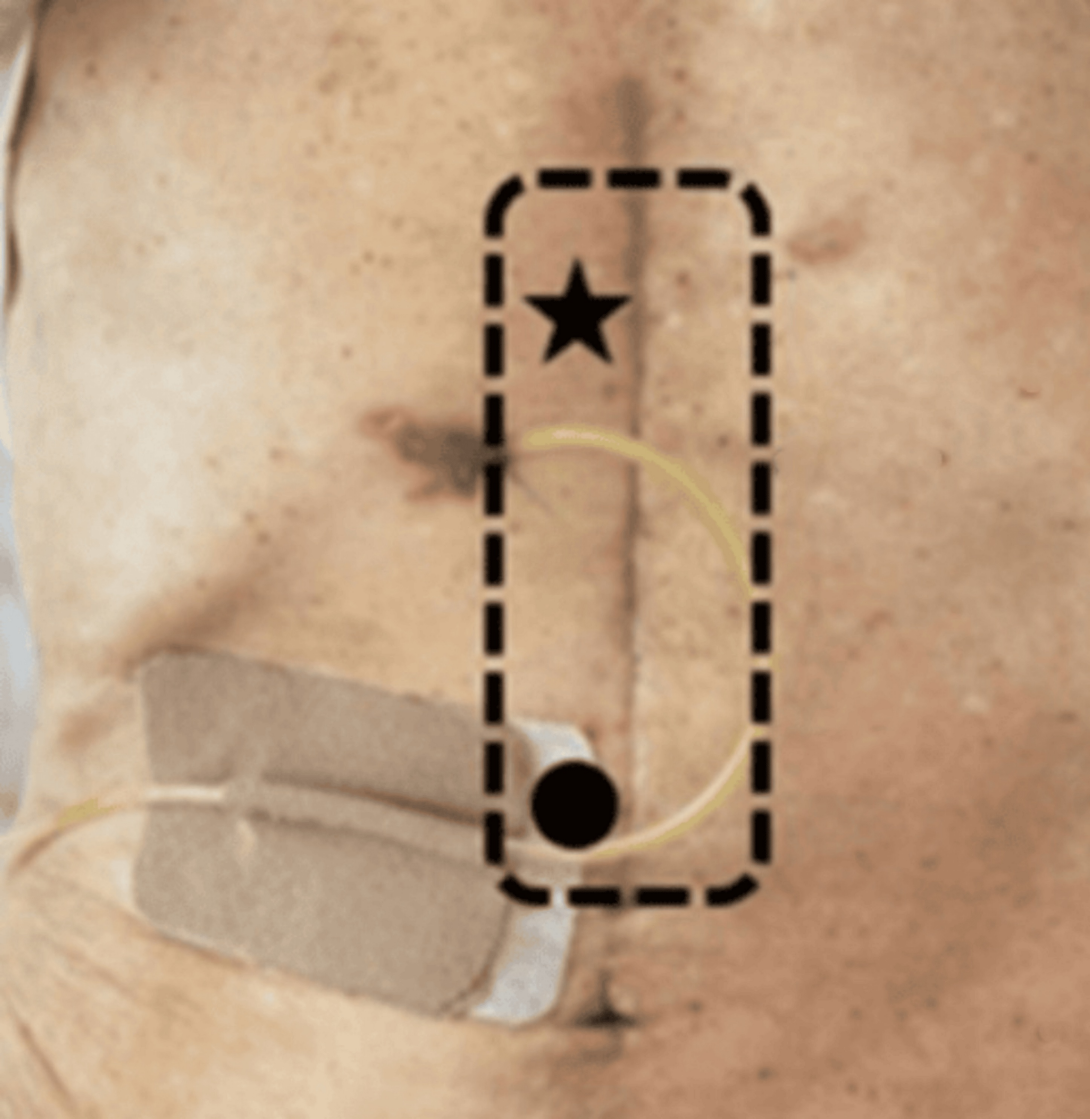
Locations of pain at the initial visit (●) and at the second visit (★). The area marked with dotted line indicates the range of dynamically shifting pain observed bilaterally along the rectus abdominis muscle at the Th7-Th9 levels developed after the recurrence of the cancer

The patient presented with pinpoint tenderness with a rating of 7/10 on the Numerical Rating Scale (NRS), localized along the medial border of the right rectus abdominis muscle at the Th9 level, adjacent to the midline surgical scar (indicated by a black dot in Figure 1). Carnett’s sign was positive, and ultrasonography (SONIMAGE MX1 with a linear probe L11-3; Konica Minolta, Tokyo, Japan) revealed a hyperechoic area in the corresponding region. A trigger point injection (TPI) with 2 mL of 1% lidocaine was administered at the site, resulting in immediate and marked pain relief. Based on these findings, a diagnosis of AMPS was made. The pain resolved significantly with a single TPI, and the patient remained symptom-free for a period thereafter. However, 131 days later, the patient again presented with sudden-onset abdominal pain, this time localized at the Th7 level of the right rectus abdominis near the surgical scar (indicated by an asterisk in Figure 1), with an NRS score of 7/10. Ultrasonography again revealed a hyperechoic lesion at the site (Figure 2). A repeat TPI was administered, resulting in effective pain relief. Notably, the patient exhibited persistent abdominal wall muscle tension, with a forward-flexed posture since his initial visit to our pain clinic. Therefore, we recommended adjunct therapy, including stretching exercises and massage therapy.

**Figure 3:**
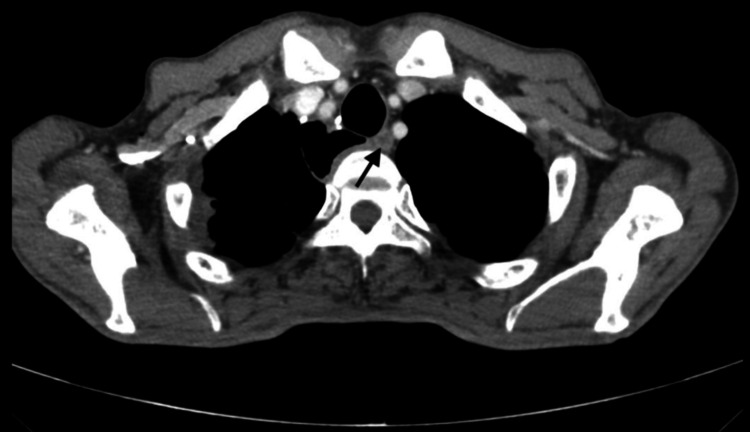
Axial CT image demonstrating a recurrent mediastinal lymph node (106recL) (arrow) CT: computed tomography

Two months later, a recurrence of mediastinal lymph node metastasis was confirmed (Figure 3), and concurrent chemoradiation therapy was initiated. Around that time, the patient began complaining of shifting pain at the Th7-Th9 levels bilaterally along the rectus abdominis (Figure 1). He was followed up biweekly, with TPI administered as requested. The analgesic effect of the TPIs gradually diminished, and the patient became increasingly bedridden, making outpatient visits difficult. Eventually, his pain management was transitioned to pharmacological therapy with tramadol and amitriptyline. As the patient’s depressive symptoms had progressively worsened, a psychiatric consultation was requested, and mirtazapine was initiated. The patient’s Eastern Cooperative Oncology Group performance status was 2 at the initial evaluation and later declined to 3, indicating progressive physical deconditioning. Four months later, due to further progression of mediastinal disease, best supportive care was initiated by the primary oncology team. The patient was subsequently transferred to a palliative care facility.

## Discussion

Epidemiology of AMPS

Among patients with advanced cancer, AMPS has been reported in approximately 40% of cases, with about 5% of TPs localized in the abdominal region [5]. Although the exact incidence of AMPS following abdominal surgery remains unclear, it is believed to result primarily from the development of myofascial TPs within the abdominal wall muscles in response to surgical trauma [1]. Given its under-recognition and diagnostic challenges, the true incidence of AMPS following abdominal surgery may be higher than currently estimated.

Etiology of AMPS

While the exact etiology of AMPS remains uncertain, the development of TPs is thought to result from multiple contributing factors, including direct injury to muscles and fascia, scar formation during tissue repair, postoperative movement restrictions, malnutrition, and persistent chronic inflammation due to impaired immune function [3]. Various medical interventions may also contribute to the development of AMPS by disrupting postural balance and increasing muscular load, particularly in patients with advanced cancer. Additionally, prolonged immobility and malnutrition may also contribute to the chronicity of symptoms [6]. Although the sudden onset of AMPS during the subacute postoperative phase (following abdominal surgery) is considered rare, the combination of surgical trauma and cancer-related pathologies may have acted synergistically in the development of AMPS in the present case.

Diagnosis of AMPS

The differentiation between AMPS and visceral pain is generally straightforward; however, AMPS is often mistaken for another type of abdominal wall pain, anterior cutaneous nerve entrapment syndrome (ACNES). Both conditions commonly present with unremarkable blood tests and imaging findings, along with a positive Carnett’s sign. On ultrasound, TPs may appear as either hypoechoic or hyperechoic areas, and hyperechoic points have also been reported in ACNES [7,8]. Therefore, differentiating between the two conditions based solely on the ultrasound imaging remains inconclusive. Nevertheless, AMPS and ACNES differ significantly in terms of pain localization and underlying pathophysiology. AMPS involves the formation of TPs at various sites within the rectus abdominis muscle; thus, pain could occur anywhere along the muscle. In contrast, ACNES results from entrapment of the anterior cutaneous branch of the spinal nerve near the lateral edge of the rectus abdominis, leading to pain that is focused within a small area (~2 cm²). In the present case, the TP was located along the medial border of the rectus abdominis, supporting the diagnosis of AMPS. However, following abdominal surgery, surgical trauma may occasionally induce TP formation near the incision site that mimics ANCES, thereby complicating the differential diagnosis [9].

Treatment of AMPS

Accurate recognition of AMPS is essential for effective pain management. TPIs are generally effective for both AMPS and ACNES. While neurectomy may be considered in refractory cases of ACNES, it is not applicable to AMPS. Therefore, careful differential diagnosis is critical when considering surgical intervention.

In the present case, however, the efficacy of TPIs gradually diminished over time. Although the efficacy of single TPI in AMPS has been demonstrated in several studies, the therapeutic value of repeated TPIs in cases with prolonged symptoms remains uncertain. Moreover, patients with elevated depressive symptoms, as indicated by high scores on the Hospital Anxiety and Depression Scale, have been reported to experience worsening of pain following TPI [4]. This association between AMPS and depression might have contributed to the development of persistent, TPI-refractory pain observed in the present case.

In addition, various non-pharmacological treatments have been explored in the management of AMPS, including physical therapy, acupuncture, dry needling, botulinum toxin injection, fascial plane blocks, specific nerve blocks, electrical nerve stimulation, low-level laser therapy, and extracorporeal shock wave therapy; however, the efficacy of these modalities warrants further investigation [10,11]. The use of strong opioids for AMPS may not only be ineffective but can also lead to distressing side effects, such as delirium and excessive sedation [12]. Therefore, distinguishing AMPS from cancer-related pain is also crucial. When a cancer patient presents with pain, the possibility of coexisting AMPS should be considered alongside tumor-related causes, and treatment should be selected accordingly.

## Conclusions

In summary, the present case highlights the importance of recognizing AMPS as a potential cause of sudden worsening of postoperative pain following abdominal cancer surgery. It also emphasizes the diagnostic and therapeutic challenges of managing AMPS in debilitated patients with advanced cancer. This case highlights the educational value of recognizing AMPS in postoperative settings, helping clinicians identify and manage similar cases effectively.
